# Persistence of Structural Changes at the Anterior Cornea in Bullous Keratopathy Patients after Endothelial Keratoplasty

**DOI:** 10.1371/journal.pone.0074279

**Published:** 2013-09-16

**Authors:** Naoyuki Morishige, Naoyuki Yamada, Yukiko Morita, Kazuhiro Kimura, Koh-Hei Sonoda

**Affiliations:** Department of Ophthalmology, Yamaguchi University Graduate School of Medicine, Ube, Yamaguchi, Japan; Cedars-Sinai Medical Center; UCLA School of Medicine, United States of America

## Abstract

Subepithelial fibrosis (SEF) and the transdifferentiation of keratocytes into fibroblasts or myofibroblasts (Fbs/MFbs) have been detected in the cornea of individuals with bullous keratopathy. We examined the anterior cornea of bullous keratopathy patients for such changes after Descemet’s stripping automated endothelial keratoplasty (DSAEK). Twenty-two individuals who underwent unilateral DSAEK at Yamaguchi University Hospital were enrolled in the study. The subjects were divided into groups A (*n* = 10) and B (*n* = 12) with a preoperative duration of stromal edema of less than or at least 12 months, respectively. The structure of the anterior stroma was examined by in vivo laser confocal microscopy at various times after surgery. SEF was detected in 1 (10.0%) and 11 (91.7%) cases in groups A and B, respectively, before surgery as well as in 0 (0%) and 7 (58.3%) cases, respectively, at 6 months after DSAEK. Fb/MFb transdifferentiation was detected in 0 (0%) and 8 (66.7%) cases in groups A and B, respectively, before surgery as well as in 0 and 1 (8.3%) case, respectively, at 6 months postsurgery. Anterior stromal scattering (ASS) was detected in 10 (100%) and 12 (100%) cases in groups A and B, respectively, before surgery as well as in 0 (0%) and 6 (50.0%) cases, respectively, at 6 months after DSAEK. Changes in anterior stromal structure apparent before surgery were thus also detected in bullous keratopathy patients after DSAEK. SEF and ASS persisted for more than 6 months in a substantial proportion of individuals with a preoperative duration of stromal edema of at least 12 months.

## Introduction

Endothelial keratoplasty such as Descemet’s stripping automated endothelial keratoplasty (DSAEK) [1-4] or Descemet’s membrane endothelial keratoplasty [5-7] is being increasingly adopted for the surgical treatment of bullous keratopathy, with the number of such procedures performed now being greater than that for penetrating keratoplasty in some countries [8,9]. The outcome and complications of endothelial keratoplasty have been compared with those for penetrating keratoplasty, with several advantages of endothelial keratoplasty having been identified, including improved visual acuity [1,7], a low frequency of graft rejection [10,11], and increased structural rigidity of the cornea due to the small size of the incision. A major difference between the two types of surgery is that penetrating keratoplasty replaces the entire corneal stroma whereas endothelial keratoplasty does not. The visual outcome of endothelial keratoplasty is thus dependent on the transparency of the corneal stroma of the patient.

In a follow-up to previous pathological studies [12,13], we have recently investigated structural alterations in the cornea of individuals with bullous keratopathy. Our observations revealed a structural abnormality beneath the basal cell layer of the corneal epithelium, designated subepithelial fibrosis (SEF) [14], that was detectable by second harmonic generation imaging microscopy [15,16]. We also found that keratocytes in the corneal stroma had undergone transdifferentiation into fibroblasts (Fbs) or myofibroblasts (MFbs) [15-17]. In addition, whereas the structure of collagen lamellae was well maintained at the anterior stroma [15], it was aberrant at the posterior stroma [16]. Pathological changes similar to these detected by laboratory analysis have been observed by in vivo confocal microscopy in the cornea of patients with bullous keratopathy [18,19] or Fuchs dystrophy [20] after DSAEK. A common factor related to the expression of these pathological changes as well as to postoperative visual acuity after DSAEK in bullous keratopathy cases was found to be the duration of stromal edema, which can lead to the development of Descemet’s folds or epithelial edema [15-17,19]. The presence of such pathological structural alterations in individuals with bullous keratopathy is thus an important consideration with regard to the performance of DSAEK surgery.

Whether endothelial keratoplasty or penetrating keratoplasty is the best surgical approach for bullous keratopathy patients needs to be determined. If structural alterations at the anterior stroma are present in such patients, their likely persistence or duration after endothelial keratoplasty is an important factor in the suitability of this procedure. We have therefore now investigated the existence and duration of structural abnormalities at the anterior stroma of bullous keratopathy patients after DSAEK surgery with the use of in vivo confocal microscopy. We found that SEF and Fb/MFb transdifferentiation were detectable after surgery, especially in individuals with a long preoperative duration of stromal edema, and that the frequency of these signs decreased with time after surgery. Our results suggest that endothelial keratoplasty may be appropriate for most individuals with bullous keratopathy if the patients are followed up after surgery until their visual acuity has recovered and the anterior pathological changes have disappeared.

## Methods

### Subjects

**Figure 1:**
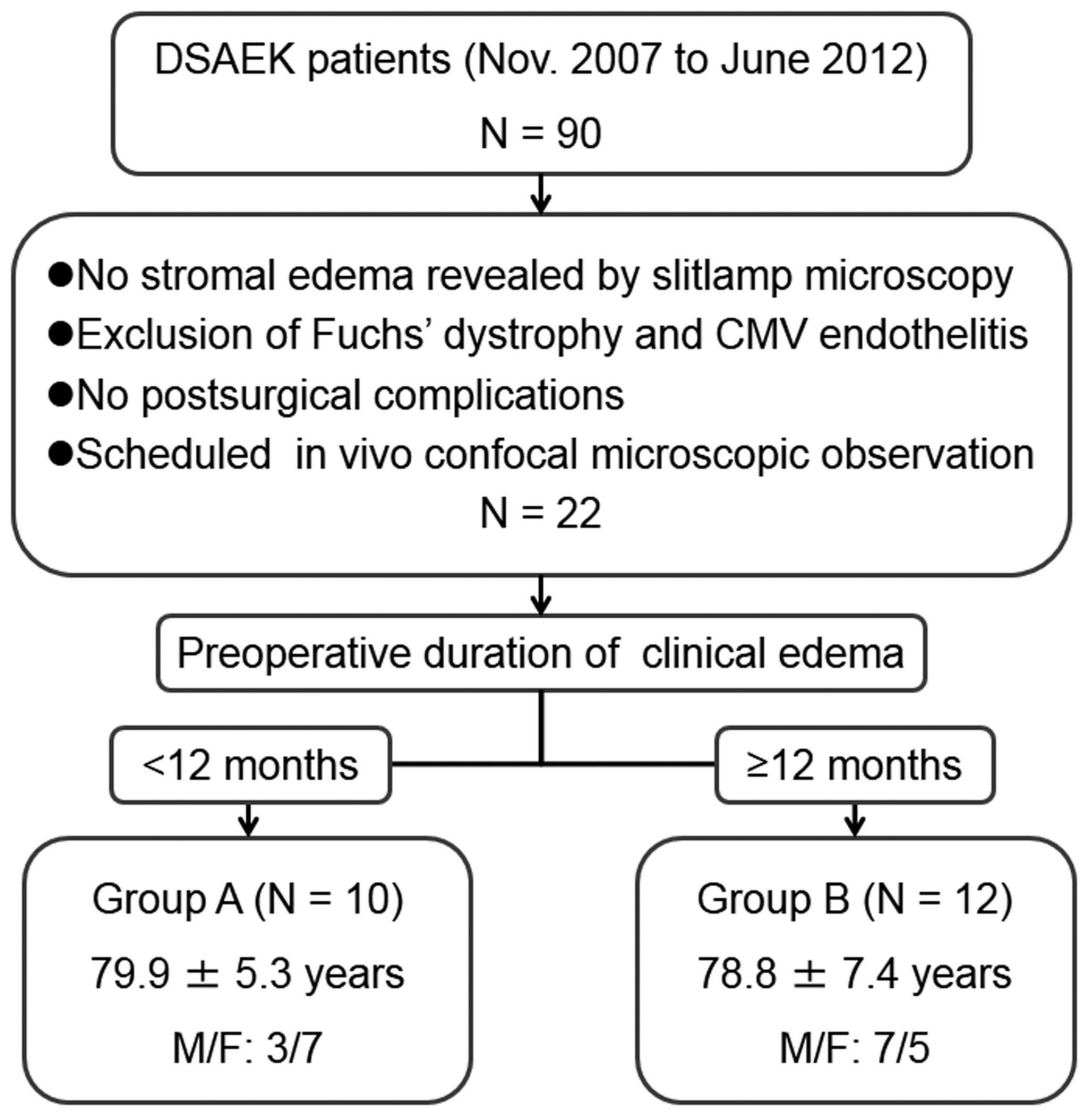
Flowchart for enrollment of study subjects.

This clinical study was approved by the Institutional Review Board of Yamaguchi University Hospital and adhered to the tenets of the Declaration of Helsinki. It was designed as a retrospective case-control study. A total of 90 patients underwent unilateral DSAEK at Yamaguchi University Hospital between November 2007 and June 2012. The patients who fulfilled the following conditions were enrolled in the study (Figure 1): (i) the corneal stroma was not clinically edematous after DSAEK as revealed by slitlamp microscopy; (ii) Fuchs’ corneal endothelial dystrophy and cytomegaloviral endothelitis were excluded as causative diseases; (iii) there were no apparent postsurgical complications; and (iv) the patients underwent scheduled confocal microscopy before as well as 1, 3, 6, and 12 months after surgery. Twenty-two individuals were thus enrolled and provided written informed consent to participation in the study. The subjects were divided into two groups on the basis of whether the preoperative duration of stromal edema was less than 12 months (group A: three men and seven women, with a mean ± SEM age at the time of surgery of 79.9 ± 5.3 years and a range of 73 to 88 years) or at least 12 months (group B: seven men and five women, with a mean ± SEM age of 78.8 ± 7.4 years and a range of 68 to 89 years). The preoperative duration of stromal edema was determined from clinical charts as the interval between the onset of clinical findings indicative of stromal edema (such as increased corneal thickness, Descemet’s folding, or epithelial edema) and the day of DSAEK surgery, as previously described [19]; the mean ± SEM values were 6.3 ± 3.2 months (range, 1 to 10) and 24.1 ± 10.6 months (range, 12 to 50) in groups A and B, respectively. Characteristics of the subjects including the underlying causes of bullous keratopathy and endothelial cell density during the observation period are shown in Table 1.

**Table 1:** Characteristics of the study subjects.

Group	No.	Age (years)/gender	Causative disease	Edema duration (months)	Endothelial cell density (cells/mm^2^)
A	1	79/F	PBK	3	2165-1282
	2	74/F	PBK	7	2232 - 1463
	3	78/F	PBK	7	1919-1368
	4	73/M	PBK	1	1733 - 1175
	5	75/F	Post-PK	9	1799 - 1751
	6	85/F	PBK	8	1709-1688
	7	86/F	PBK	2	2016 - 1002
	8	78/F	PBK	10	1302 - 932
	9	83/M	PBK	9	2725 - 1180
	10	88/M	PBK	7	2212 - 1201
B	1	83/F	PBK	30	2801 - 1898
	2	82/M	PBK	27	1527 - 1063
	3	68/F	PBK	30	2037-1499
	4	86/F	PBK	23	2500 - 832
	5	87/F	PBK	12	1881 - 1422
	6	68/M	PBK	27	1718-1458
	7	83/F	PBK	26	2427 - 2331
	8	71/M	PBK	14	2717 - 1241
	9	76/M	Post-PK	50	2717 - 1658
	10	73/M	Post-PK	16	2985 - 1435
	11	80/M	PBK	22	1757-1299
	12	89/M	PBK	12	1783 - 1082

Abbreviations: PBK, pseudophakic bullous keratopathy; PK, penetrating keratoplastyValues for endothelial cell density shows the range of the endothelial density after DSAEK surgeries.

### In vivo laser confocal microscopy

In vivo laser confocal microscopy with a Heidelberg Retina Tomography II–Rostock Corneal Module (Heidelberg Engineering, Heidelberg, Germany) was performed before as well as 1, 3, 6, and 12 months after surgery, as we previously described [19]. In brief, the eye was subjected to topical anesthesia and coupling gel (Comfort gel; Dr. Mann Pharma, Berlin, Germany) was applied to the surface of the objective lens cone, which was then carefully positioned at the central area of the cornea. We were able to observe the entire cornea, from the surface of the epithelium to the endothelium, and we collected more than 100 JPEG images of 384 by 384 pixels. Eye movement was monitored during the examination period, and we confirmed that all images were obtained from the central area of the cornea. A clinical technician selected the images derived from the basal cell layer, Bowman’s layer, and anterior stroma in a blinded manner. A second clinical technician then selected images determined to be representative of these three regions for each subject, and a third clinical technician determined whether the selected images showed a normal or abnormal structure, again in a blinded manner.

## Results

**Figure 2:**
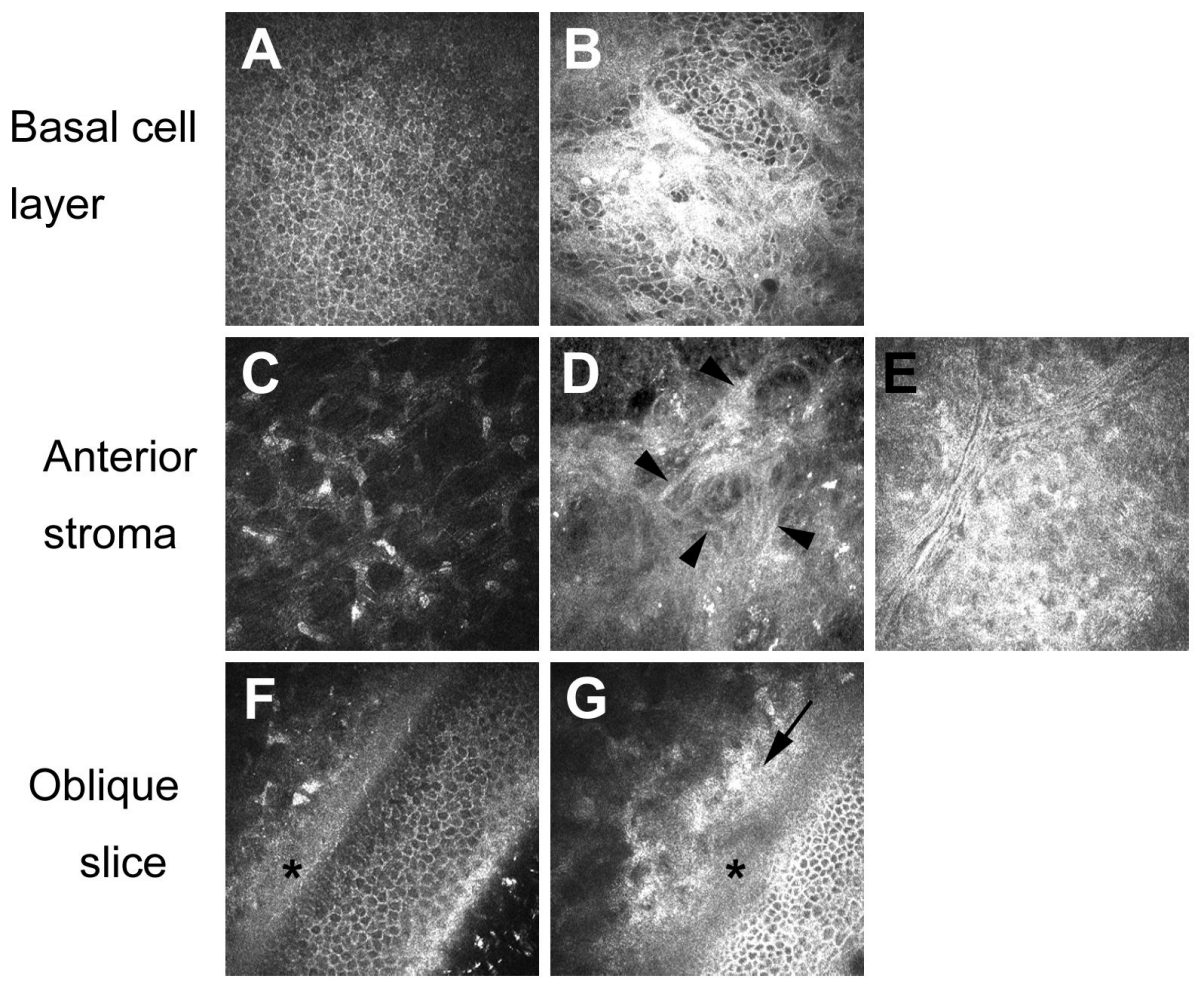
Representative confocal images of the cornea after DSAEK surgery. (A) Normal appearance of the basal cell layer of the corneal epithelium. (B) Subepithelial fibrosis (SEF) at the level of the basal cell layer. (C) Normal appearance of anterior stromal keratocytes. (D) Fibroblast or myofibroblast (Fb/MFb) transdifferentiation of keratocytes in the anterior stroma. Arrowheads indicate fibrous structures of the fibroblasts or myofibroblasts. (E) Anterior stromal scattering (ASS). ASS is characterized by a higher level of scattering and difficulty in identification of cell nuclei. (F) Normal appearance of the anterior cornea in an oblique view. The asterisk indicates Bowman’s layer. (G) Abnormal appearance of the anterior cornea in an oblique view. The asterisk and arrow indicate Bowman’s layer and ASS (located beneath Bowman’s layer), respectively.

In vivo confocal microscopy revealed either a normal structure for the basal cell layer of the corneal epithelium (Figure 2A) or the presence of subepithelial fibrosis (SEF) (Figure 2B) in the study subjects after DSAEK. Similarly, keratocytes at the anterior stroma either showed a normal structure (Figure 2C) or manifested increased intensity of cellular components including apparently fibrous structures, indicating that they had undergone transdifferentiation into fibroblasts or myofibroblasts (Fbs/MFbs) (Figure 2D). In addition, highly scattering lesions in which it was difficult to identify cell nuclei were detected at the anterior stroma (Figure 2E). These lesions, which we designated anterior stromal scattering (ASS), were similar to the subepithelial haze described previously [18]. Oblique confocal images also revealed a normal structure of the anterior cornea (Figure 2F) or the spatial localization of ASS (Figure 2G). On the basis of these observations, we determined the frequencies of SEF, Fb/MFb transdifferentiation, and ASS as abnormal structural changes.

**Figure 3:**
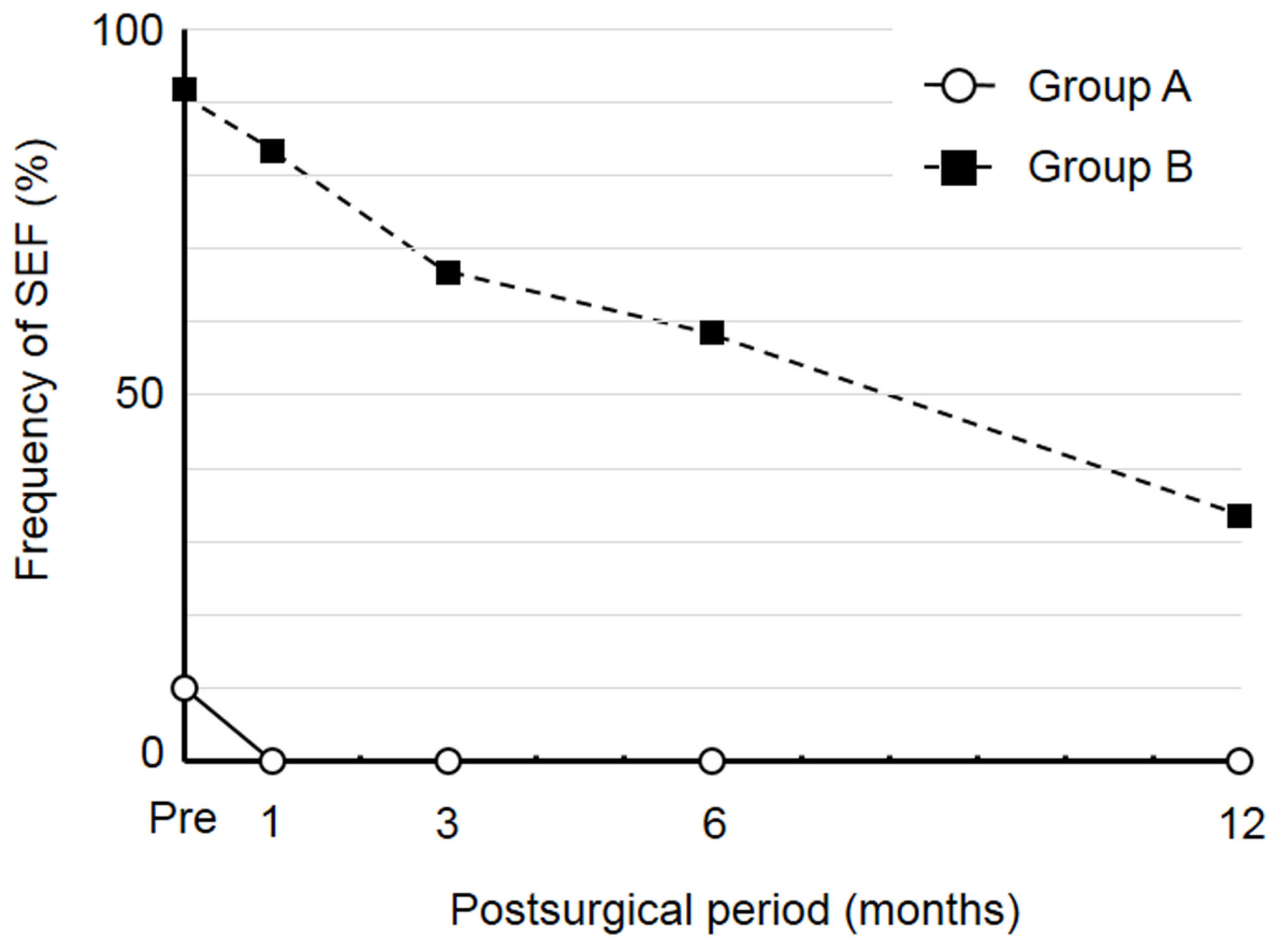
Frequency of SEF in groups A and B before and after DSAEK.

**Figure 4:**
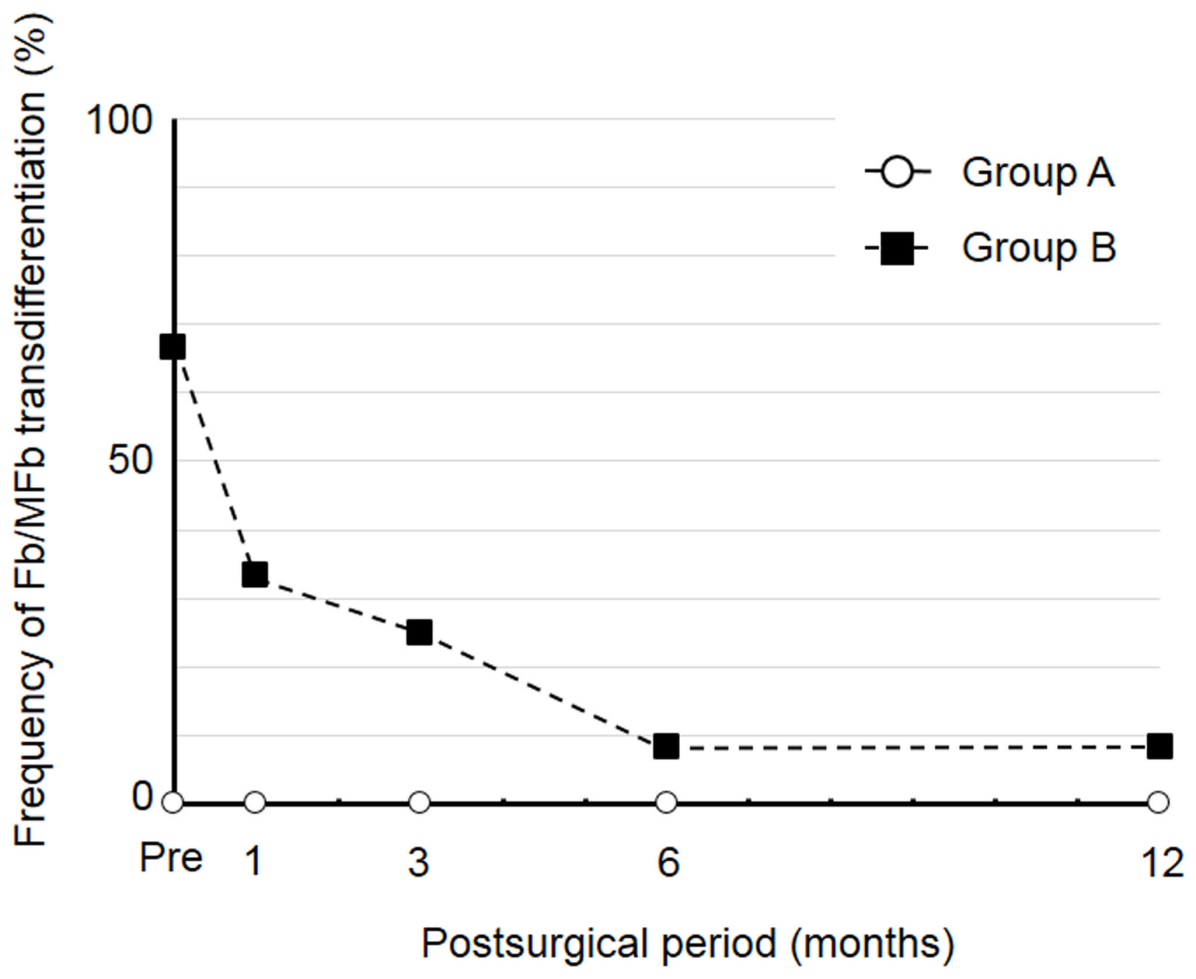
Frequency of Fb/MFb transdifferentiation in groups A and B before and after DSAEK.

**Figure 5:**
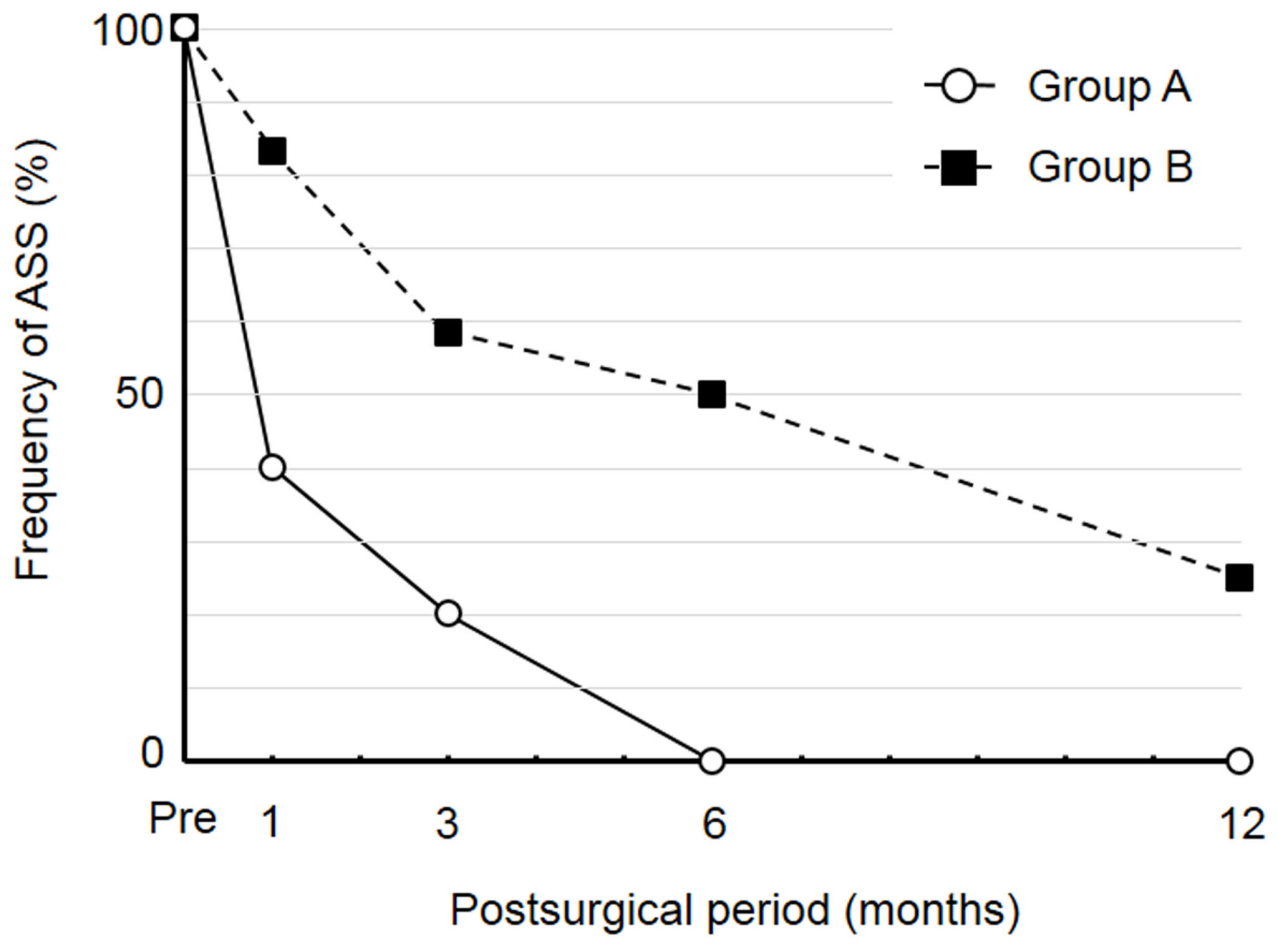
Frequency of ASS in groups A and B before and after DSAEK.

SEF was detected in 1 (10.0%) and 0 (0%) of the 10 cases in group A before and at all times after DSAEK, respectively, as well as in 11 (91.7%), 10 (83.3%), 8 (66.7%), 7 (58.3%), and 4 (33.3%) of the 12 cases in group B before and 1, 3, 6, and 12 months after DSAEK, respectively (Figure 3). Fb/MFb transdifferentiation was not detected before or after DSAEK in group A but was apparent in 8 (66.7%), 4 (33.3%), 3 (25.0%), 1 (8.3%), and 1 (8.3%) of the 12 cases in group B before and 1, 3, 6, and 12 months after DSAEK, respectively (Figure 4). ASS was detected in 10 (100.0%), 4 (40.0%), 2 (20.0%), 0 (0%), and 0 (0%) of the 10 cases in group A as well as in 12 (100%), 10 (83.3%), 7 (58.3%), 6 (50.0%), and 3 (25.0%) of the 12 cases in group B before and 1, 3, 6, and 12 months after DSAEK, respectively (Figure 5).

## Discussion

We have here shown that pathological alterations such as SEF, Fb/MFb transdifferentiation, and ASS were detected by in vivo confocal microscopy in individuals with bullous keratopathy after DSAEK surgery. ASS was the most frequent abnormality, being present in all subjects before surgery, but its frequency decreased relatively rapidly after DSAEK. Indeed, the frequency of all structural abnormalities declined with time after surgery. However, the frequency of each abnormality after surgery remained higher in individuals with a preoperative duration of stromal edema of at least 12 months than in those with a duration of less than 12 months.

Consistent with previous observations [19,20], with the use of in vivo confocal microscopy we detected changes in anterior stromal structure both before and after DSAEK. The nature of the structural changes detected and their relation to the preoperative duration of stromal edema were similar to those described in our previous studies [15-17]. SEF was detected frequently, especially in group B (longer preoperative duration of stromal edema). This structural change results from the abnormal accumulation of extracellular matrix [13,14], and the slow recovery rate observed for this change likely reflects the time required for degradation of the aberrant matrix. In contrast, although the Fb/MFb phenotype was also frequently detected in group B, it disappeared rapidly after DSAEK. Fbs and MFbs are formed through transdifferentiation of keratocytes [21], and it is possible that Fbs revert back to keratocytes and that MFbs are eliminated. ASS was the most frequently detected abnormality in both subject groups in the present study, being present in all individuals before surgery. We previously showed that the signal intensity for the anterior stroma as detected by in vivo confocal microscopy is greater for bullous keratopathy patients than for normal individuals [22]. It is possible that the increased ASS in the bullous keratopathy cornea reflects the increased water content of the tissue, in which case the rapid decrease in the frequency of ASS observed in group A would represent a rapid reduction in the water content of the edematous stroma. It is also possible, however, that opaque structural abnormalities such as SEF and Fbs/MFbs contribute to ASS. The slow decline in the frequency of SEF in group B may thus account in part for the persistence of ASS in this group. Overall, our results suggest that the observed stromal pathological changes may be a reversible consequence of stromal edema, with the extent of the changes decreasing after elimination of the edema.

We have applied in vivo laser confocal microscopy to detect preoperative and postoperative structural abnormalities of the cornea in individuals with bullous keratopathy. The advantage of this approach is that it is noninvasive and therefore allows for repeated observation of corneal structure in a given patient. A related disadvantage is that it is difficult to observe the same precise lesions at different observation times because of the small area (~400 µm^2^) of most lesions and because of small eye movements of the patient. The determination of lesion frequency in a group of patients over time may thus not be without potential error. Nevertheless, we believe that our present results are valid, given the similarities in the lesions among patients and the similar patterns of their disappearance after DSAEK. Our data thus indicate that SEF, Fbs/MFbs, and ASS disappear after DSAEK surgery as a result of the attenuation of corneal stromal edema. Another potential limitation of our study is the assumption that the structural changes detected by in vivo confocal microscopy are related to pathological changes characterized previously in the laboratory. In this regard, our unpublished observations indicate that the structural characteristics of SEF, which is localized within or beneath the basal cell layer of the corneal epithelium and consists of the accumulation of collagen and Fbs/MFbs, are highly similar between in vivo confocal images and images obtained from the same cornea by combined immunofluorescence and second harmonic generation microscopy (Figures S1 and S2). The characteristics of Fbs/MFbs at the anterior stroma as revealed by in vivo confocal microscopy are also highly similar to those detected by immunofluorescence microscopy [16,17]. On the other hand, it has been difficult to demonstrate ASS by an imaging method other than in vivo confocal microscopy. As we mention above, we speculate that ASS might be directly attributable to an increased water content of the anterior stroma, which would not be detectable by immunofluorescence microscopy or second harmonic generation imaging microscopy.

We previously showed that visual acuity after DSAEK surgery in bullous keratopathy patients with a short preoperative duration of stromal edema was significantly greater than that for those with a long such duration [19]. Individuals with other ocular conditions that affect visual acuity, such as macular disease, advanced glaucoma, and irregular astigmatism, were excluded from this previous study in order to reveal the relation between the preoperative duration of stromal edema and postoperative visual acuity. In the present study, individuals with other ocular diseases were not excluded, and so we were not able to address such a relation. Another previous study [23] and our clinical experience suggest that postoperative visual acuity improves gradually and has largely recovered by 3 to 6 months after DSAEK surgery. The overall evidence thus indicates that postoperative visual acuity may be affected by the presence of structural changes at the anterior stroma.

A recent study showed that phototherapeutic keratectomy was effective for the treatment of anterior stromal haze after DSAEK [24]. Our previous results have suggested that pathological changes at the anterior stroma in bullous keratopathy patients affect visual acuity after DSAEK [19]. Removal of the hazy anterior stroma in bullous keratopathy patients with a long preoperative duration of stromal edema by phototherapeutic keratectomy after DSAEK is thus a possible approach to improve postoperative visual acuity. However, our observations in the present study indicate that pathological changes at the anterior cornea in such patients disappear with time after DSAEK. Removal of the anterior stroma by phototherapeutic keratectomy might thus be best reserved as an option after observation of the anterior stroma by in vivo confocal microscopy and evaluation of visual acuity at several months after DSAEK.

We have found that structural changes at the anterior stroma of bullous keratopathy patients can remain months after DSAEK surgery, with the frequency of such changes being greater in individuals with a long preoperative duration of stromal edema at all times after DSAEK. This relation is likely to be more of an issue in countries with a limited supply of donor corneas, in that bullous keratopathy patients may have to wait for a substantial period before the opportunity for keratoplasty arises. In addition to our previous and present findings, another group recently found that pathological changes such as formation of deep stromal scars, neovascularization, and inflammatory cell infiltration were more frequent in patients with a duration of bullous keratopathy of more than 1 year [25]. The preoperative duration of bullous keratopathy thus appears to be an important determinant of pathological changes in the structure of the corneal stroma. The success of endothelial keratoplasty is dependent on the transparency of the corneal stroma of the patient. Corneal surgeons should thus be aware that the preoperative duration of stromal edema is a factor that might affect the outcome of endothelial keratoplasty and should therefore be taken into account in the timing of surgery.

## Supporting Information

Figure S1 **Images of SEF obtained by in vivo confocal microscopy and by combined immunofluorescence and second harmonic generation (SHG) imaging microscopy for the same patient with bullous keratopathy.** (**A**) In vivo confocal microscopy. Fibroblastic cells were observed. The asterisk indicates basal cells, confirming that the optical slice was located in the basal cell layer of the corneal epithelium and not in the anterior stroma. (**B**) Immunofluorescence–SHG imaging microscopic analysis of a frontal slice of the cornea obtained at the time of penetrating keratoplasty. Green, α-smooth muscle actin (αSMA); blue, nuclei; magenta, SHG backward signal (collagen-derived weak signal); cyan, SHG forward signal (oriented collagen fiber–derived signal). αSMA-positive fibroblastic cells (myofibroblasts) were observed within accumulated collagen. (**C**) Sectional immunofluorescence–SHG imaging microscopic analysis, with the arrowhead indicating the location of the slice in (B). Note that αSMA-positive cells are located not underneath but above Bowman’s layer.
(TIF)

Figure S2 **Images of Fbs/MFbs obtained by in vivo confocal microscopy and by immunofluorescence–SHG imaging microscopy for the same patient with bullous keratopathy.** (**A**) In vivo confocal microscopy. Fibroblastic cells are indicated by the arrows. (**B**) Immunofluorescence–SHG imaging microscopic analysis of a frontal slice of the cornea obtained at the time of penetrating keratoplasty. Colors are as in Figure 1. αSMA-positive myofibroblasts were observed within accumulated collagen. (**C**) Sectional immunofluorescence–SHG imaging microscopic analysis, with the arrowhead indicating the location of the slice in (B). Note that αSMA-positive cells are located below Bowman’s layer.
(TIF)
