# Supplementary figures and images for: Persistence of Structural Changes at the Anterior Cornea in Bullous Keratopathy Patients after Endothelial Keratoplasty
Source: PLoS One. 2013 Sep 16;8(9):e74279. doi: 10.1371/journal.pone.0074279 (PMC3774772; doi:10.1371/journal.pone.0074279)

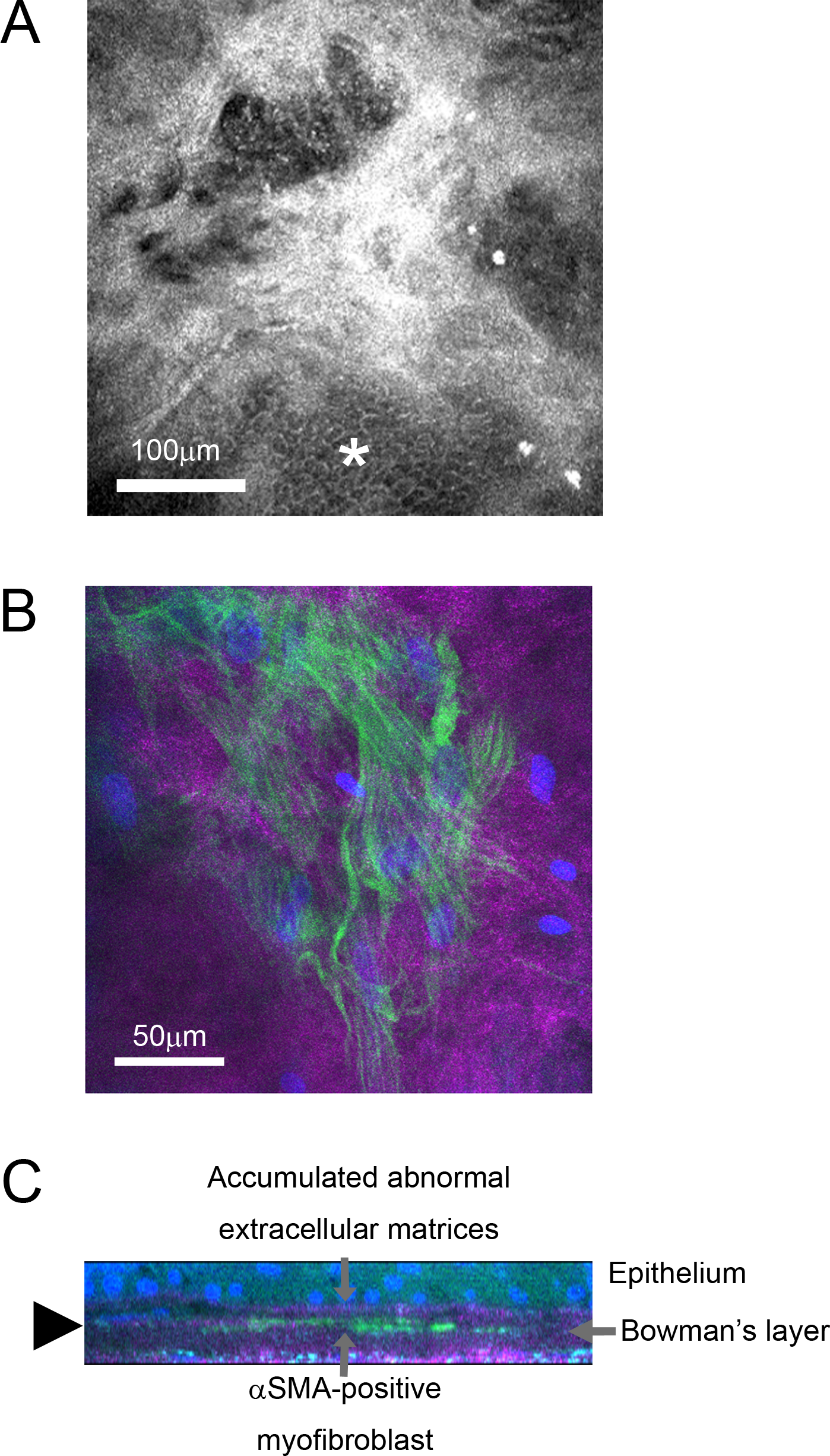

Supplement: Figure S1 — Images of SEF obtained by in vivo confocal microscopy and by combined immunofluorescence and second harmonic generation (SHG) imaging microscopy for the same patient with bullous keratopathy. (A) In vivo confocal microscopy. Fibroblastic cells were observed. The asterisk indicates basal cells, confirming that the optical slice was located in the basal cell layer of the corneal epithelium and not in the anterior stroma. (B) Immunofluorescence–SHG imaging microscopic analysis of a frontal slice of the cornea obtained at the time of penetrating keratoplasty. Green, α-smooth muscle actin (αSMA); blue, nuclei; magenta, SHG backward signal (collagen-derived weak signal); cyan, SHG forward signal (oriented collagen fiber–derived signal). αSMA-positive fibroblastic cells (myofibroblasts) were observed within accumulated collagen. (C) Sectional immunofluorescence–SHG imaging microscopic analysis, with the arrowhead indicating the location of the slice in (B). Note that αSMA-positive cells are located not underneath but above Bowman’s layer. (TIF) [file pone.0074279.s001.tif]

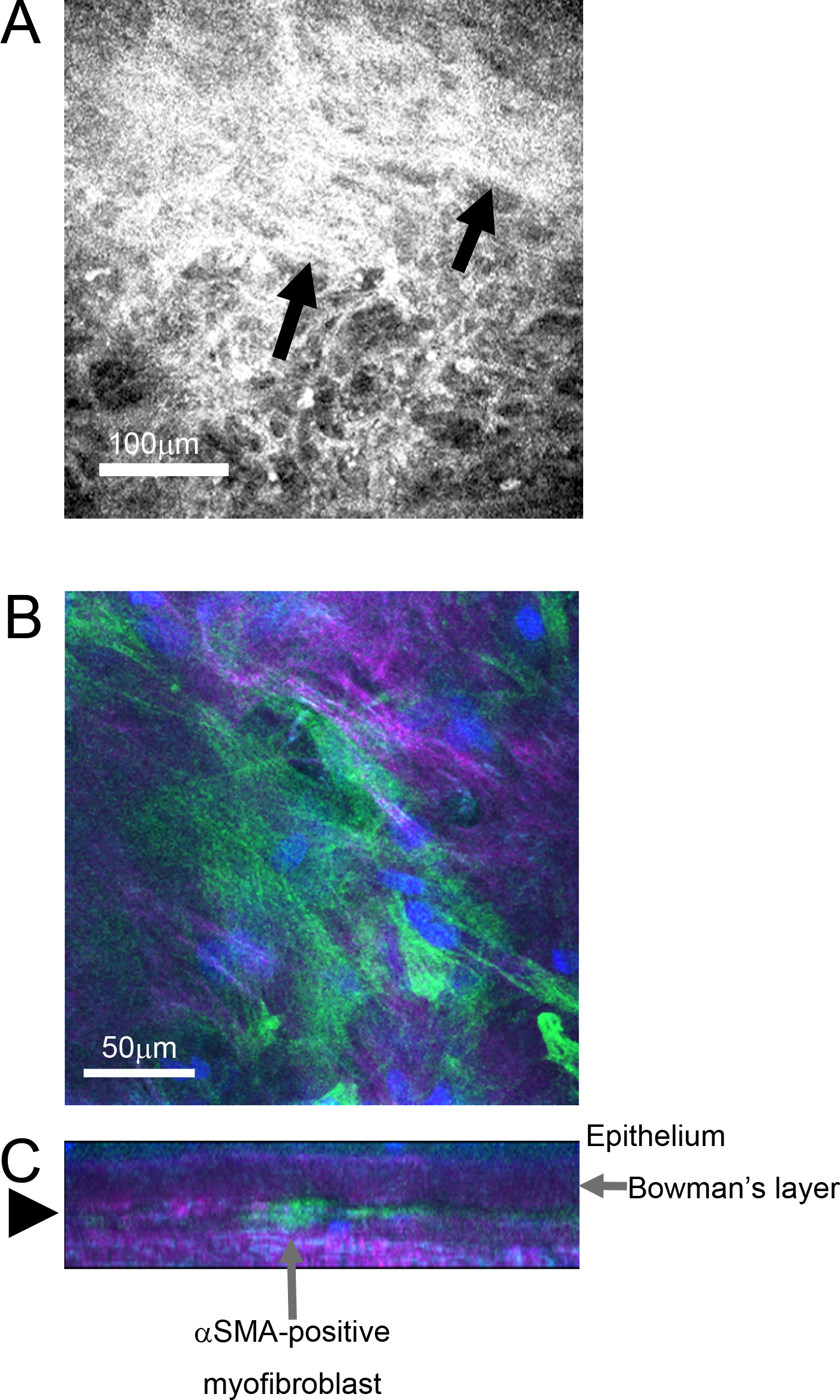

Supplement: Figure S2 — Images of Fbs/MFbs obtained by in vivo confocal microscopy and by immunofluorescence–SHG imaging microscopy for the same patient with bullous keratopathy. (A) In vivo confocal microscopy. Fibroblastic cells are indicated by the arrows. (B) Immunofluorescence–SHG imaging microscopic analysis of a frontal slice of the cornea obtained at the time of penetrating keratoplasty. Colors are as in Figure 1. αSMA-positive myofibroblasts were observed within accumulated collagen. (C) Sectional immunofluorescence–SHG imaging microscopic analysis, with the arrowhead indicating the location of the slice in (B). Note that αSMA-positive cells are located below Bowman’s layer. (TIF) [file pone.0074279.s002.tif]
